# VISIOCPR: Monocular Vision-Based CPR Training System with Human-Computer Collaborative Feedback

**DOI:** 10.3390/s26113388

**Published:** 2026-05-27

**Authors:** Ang Li, Wei Lu

**Affiliations:** School of Communications and Information Engineering, Nanjing University of Posts and Telecommunications, Nanjing 210023, China; 20210151@njupt.edu.cn

**Keywords:** cardiopulmonary resuscitation, monocular depth estimation, human–computer interaction, medical training, real-time feedback

## Abstract

High-quality cardiopulmonary resuscitation (CPR) aims at saving lives in time-critical emergencies, which requires correct compression rate, depth, and hand placement. However, due to the high cost and environmental constraints of sensor-equipped manikins or dedicated hardware, it is unrealistic to deploy these devices in ordinary training settings. For monocular vision-based methods, estimating compression depth without direct depth signals and tracking hands under severe overlap are difficult. To address these problems, this paper proposes VISIOCPR, a monocular vision-based CPR training system with human-computer collaborative feedback, which provides quantitative CPR coaching using only a standard RGB camera. To address the inherent visual constraints, the system integrates a tiered compression-point detector that maintains robust tracking continuity despite severe hand overlap and motion blur. Furthermore, it recovers accurate metric depth without attached markers through a fused calibration scheme, which combines an empirical baseline, a reference-object measurement, and visible body proportions. A randomized controlled study (n=40) showed that participants trained with VISIOCPR achieved higher simultaneous compliance and reached competency faster than the control group under the tested setting.

## 1. Introduction

Out-of-hospital cardiac arrest remains a time-critical emergency, and the opportunity for effective intervention is short [1,2,3]. Immediate cardiopulmonary resuscitation (CPR) from people nearby can significantly improve survival. However, this only works when compressions are delivered within an acceptable range of rate, depth, and hand placement. Although the American Heart Association defines that range explicitly [4], many learners practise without any source of real-time correction. This lack of feedback negatively affects overall training outcomes. Specifically, a trainee may remember the general procedure and still miss the quantitative targets that determine whether the manoeuvre is effective. Therefore, the practical issue is not whether feedback is useful, but how to provide it in ordinary training settings.

Existing solutions have shown that real-time feedback improves CPR quality during practice. However, they heavily rely on equipment that is rare outside formal training environments. On the one hand, accelerometer-based systems and force-sensing pads provide reliable rate and depth estimates [5,6,7]. Marker-based vision systems can also achieve high precision in controlled settings [8]. The trade-off for these systems is practical rather than conceptual, as these methods depend on dedicated devices, consumables, or complex setup routines. Many homes, schools, and community settings simply lack these specialized resources. On the other hand, depth cameras reduce this burden but still introduce extra hardware and environmental constraints. They often struggle in spaces with unstable lighting or reflective surfaces [9]. Compared to them, recent RGB-video approaches remove the hardware barrier [10,11], yet they mostly classify errors qualitatively. For effective coaching, qualitative feedback is often not enough. Knowing a compression is “too shallow” is helpful, but knowing the exact deviation is more actionable.

This is the point at which monocular video becomes difficult. A single RGB stream does not contain metric depth. Therefore, compression amplitude must be inferred from image motion and tied to a scale factor. That factor is not stable by default, since it changes with camera placement, viewing distance, and lens characteristics. In addition, tracking the hands is also less straightforward than it looks. During compressions, the hands overlap, reverse direction quickly, and often blur. This makes a single detector unreliable over an entire session. In addition, position assessment introduces another related tracking problem. The system needs a reference point to judge if the hands remain over the lower sternum. This point must be anatomically meaningful and visually recoverable without attached markers. The task is therefore not only one of recognition; it is also one of calibration, continuity, and anatomical anchoring.

To overcome this dilemma, we propose VISIOCPR with these specific constraints in mind. The system uses a standard webcam and runs on consumer hardware. It cannot rely on dense depth sensing, long calibration routines, or manual annotation. Instead, the tracking process is organized as a hierarchy. Specifically, the system uses hand keypoints when they are clearly visible. If they become unstable due to overlap or blur, it switches to a motion-centroid estimate. If both cues fail, a synthetic fallback preserves interface continuity, and those frames are marked invalid for scoring. Metric depth is estimated through a fused calibration scheme. This scheme combines an empirical baseline, a reference-object measurement, and a body-proportion cue. In particular, users complete a short setup sequence before practice begins. They mark the region of interest and identify the target sternum location. The interface then displays rate, depth, and hand position continuously. It issues spoken prompts only when a deviation persists long enough to be a meaningful error.

We evaluated VISIOCPR in a randomized controlled study comparing system-guided training with instructor-led practice. We measured immediate performance after training and again at follow-up to assess retention rather than within-session improvement alone. The main contributions of this work can be summarized as follows:

- We propose a webcam-based CPR training pipeline providing continuous estimates of rate, depth, and hand position.
- We design a detection and calibration scheme tailored to the specific failure modes of monocular CPR analysis.
- A controlled evaluation showed that system-guided training improved three-metric compliance and short-term retention under the tested setup.

The remainder of this paper is organized as follows. The related work of sensor-based CPR systems, RGB and depth-camera approaches, and monocular depth estimation is introduced in Section 2. Our system design, study protocol, and empirical findings are presented in Section 3 and Section 4. Finally, the conclusion of our paper is presented in Section 5.

## 2. Related Work

### 2.1. Sensor-Based and Hardware-Dependent Systems

Early CPR feedback systems reached high accuracy by instrumenting the body, the manikin, or both. Specifically, Mini-VREM used infrared reflective markers and a dedicated camera setup to achieve high depth accuracy [8]. The limitation is not that the measurements are poor. The setup simply presumes a highly controlled training environment. Marker placement and camera alignment introduce operational overhead. This overhead is hard to justify in low-cost or unsupervised practice settings.

Accelerometer-based and force-sensing devices avoid many of the problems faced by vision systems. Published studies report accurate rate and depth estimation when motion is measured directly at the compression site [5,6,7]. At the same time, these devices observe only what their sensors are positioned to measure. They are informative about displacement and rhythm, but less informative about continuous hand placement unless extra instrumentation is added. They also assume access to dedicated hardware, which is precisely the access constraint that camera-only approaches try to relax. To provide comprehensive feedback, some studies analyze motion-capture data to extract CPR quality parameters [12]. However, the high device cost and complex setup routines hinder the widespread deployment of these sensor-based systems.

### 2.2. Vision-Based and Depth-Camera Systems

With the rapid development of computer vision, vision-based systems have become a popular alternative for CPR training. Depth cameras opened a useful middle ground between body-mounted sensors and ordinary RGB video. Systems such as RELIVE, LISSA, and AIMS track compression motion without markers using depth cameras [9,13,14]. Their main limitation is again mostly practical. A depth camera is an extra device that must be purchased and positioned. They are also sensitive to ambient infrared light and specific room layouts. For laboratory instruction this may be acceptable; for casual or repeated home use it is less attractive.

RGB-based systems are the most attractive from a deployment perspective because they rely on hardware users already own. Recent work identifies CPR errors and training phases from standard video with good expert agreement [10,11]. In addition, computer vision techniques based on modular neural networks have been applied to automatic assessment of physical therapy rehabilitation activities [15], which shares similar challenges with CPR training. Furthermore, deeply learned view-invariant features have been proposed for cross-view action recognition [16], providing potential solutions for handling different camera viewpoints. In general, vision-based systems in healthcare have shown great potential in various use cases and real-time applications [17]. What remains less developed is the recovery of continuous physical measures from that same video stream. In the context of CPR coaching, this distinction is important. A categorical judgement tells the learner that something is wrong. A metric estimate shows exactly how far the performance deviates from the recommended range.

### 2.3. Monocular Depth Estimation

The broader computer-vision literature provides a useful frame for this problem. Monocular depth estimation has improved substantially through convolutional models and transformer-based architectures [18,19]. Even so, a familiar limitation remains: depth recovered from a single image is ambiguous up to scale. If the task requires metric output rather than relative ordering, an additional reference or constraint is needed.

The CPR setting offers a narrower and, in some respects, more manageable geometry than general scene understanding. The camera is usually fixed during a session, and the motion is constrained to a limited region. Plausible scale references are also often visible in the frame. For that reason, we do not attempt to estimate full-scene depth. VISIOCPR uses local visual cues and explicit geometric assumptions to recover the scale needed for coaching. This keeps the computation light enough for real-time use on a standard laptop.

## 3. Methodology

### 3.1. System Architecture

**Figure 1:**
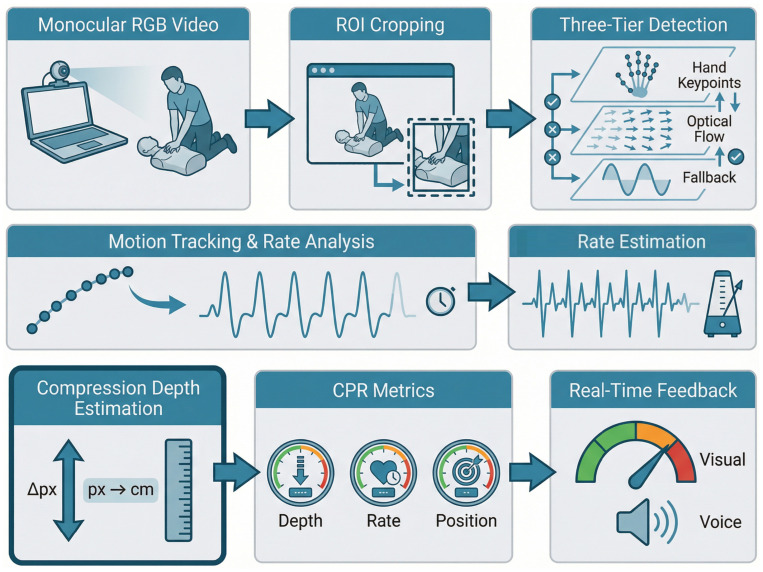
Processing pipeline of VISIOCPR. The system extracts compression metrics from monocular RGB video through three stages: region cropping and tiered detection, motion tracking and rate estimation, and fused depth calibration.

The overview of our system architecture is shown in Figure 1. The system takes monocular RGB video from a standard webcam and isolates the manikin chest through region-of-interest cropping. Then, a three-tier detection module extracts the compression trajectory, which is analyzed to estimate the compression rhythm and rate. Finally, the pixel displacement is converted into physical depth using a fused calibration scheme, allowing the system to present real-time visual and voice feedback on rate, depth, and hand position.

This architecture is designed for interactive use on a standard laptop CPU without dedicated graphics hardware. The design prioritizes tracking continuity over intermittent high-precision detections, uses redundant calibration sources to handle visual occlusions, and keeps the initial user setup under 30 s to encourage practical adoption. It is not intended as a general-purpose pose or depth model; rather, it uses the constrained geometry of CPR practice to estimate the three quantities needed by the feedback interface.

**Figure 2:**
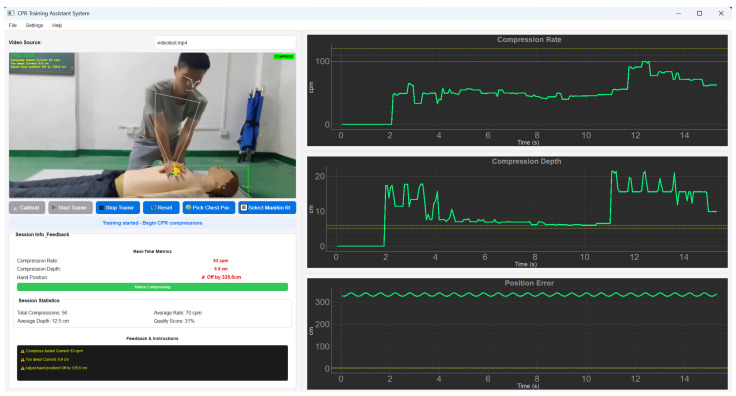
VISIOCPR software 1.0 interface. (**Left**): live camera feed with ROI boundary (green rectangle), detected compression trajectory (red trace), and sternum reference point (yellow cross). (**Right**): metric gauges for compression rate (target 100–120 cpm), depth (target 5–6 cm), and hand position error. Green indicates compliance; yellow indicates a marginal value; red indicates a guideline violation. Voice prompts are overlaid as text when a correction is warranted.

The interface is shown in Figure 2. The live video panel overlays the region of interest, the estimated compression trajectory, and the sternum reference marker. A companion panel displays rate, depth, and position error against guideline bands. Text prompts are shown when the system determines that a corrective message is warranted.

### 3.2. ROI Cropping and Three-Tier Detection

The main reason for adopting a tiered detector is that pose estimation alone is not stable enough for this task. In CPR video, the hands frequently overlap and one hand can occlude the other. The direction of motion also reverses quickly near the top and bottom of the compression cycle. In our pilot recordings, these conditions repeatedly caused landmark confidence to collapse for short intervals. To solve this issue, rather than treating those failures as exceptions, we incorporated them into the design and used explicit fallback modes.

**Region of interest.** First and foremost, before training starts, the user draws a rectangle over the manikin chest, and everything outside this box is ignored. Restricting the field of analysis reduces processing cost and lowers the chance that unrelated motion will be mistaken for chest compressions. The system checks the basic geometry of the selected box and warns the user if the region is too small or implausibly shaped.

**Tier 1: pose-based keypoints.** The primary tracker uses MediaPipe’s hand model [20] to locate three candidate points: the dominant wrist, the middle-finger joint, and the hand-region centroid. For each candidate, a composite score $c_{1}$ combines detector confidence, temporal smoothness, and agreement with a Kalman prediction:

(1) $$c_{1}=0.5\;s_{\det}+0.3\;s_{\mathrm{temp}}+0.2\;s_{\mathrm{motion}},$$

where $s_{\det}$ is the raw landmark confidence, $s_{\mathrm{temp}}$ measures smoothness through a second-order displacement difference, and $s_{\mathrm{motion}}$ reflects agreement with the predicted motion state. The weights were selected by a small grid search on held-out pilot recordings and normalized to sum to one. This procedure was used to keep the score tied to observed tracking behaviour rather than to a purely arbitrary heuristic.

**Tier 2: motion centroid.** When pose confidence falls below the switching threshold, the system switches to motion-based localization. A Gaussian mixture model separates foreground from the region of interest, and optical flow vectors are thresholded by magnitude and merged with the foreground mask. Simple morphological cleanup removes isolated noise, after which connected-component analysis identifies the dominant moving region. Its centroid becomes the compression-point estimate. The confidence score for this tier is

(2) $$c_{2}=0.4\;s_{\mathrm{stab}}+0.3\;s_{\mathrm{persist}}+0.3\;s_{\mathrm{freq}},$$

where $s_{\mathrm{stab}}$ measures mask-area consistency across recent frames, $s_{\mathrm{persist}}$ evaluates component continuity, and $s_{\mathrm{freq}}$ checks if the motion frequency is within a plausible CPR range. This last term is important because motion alone is too permissive; background disturbances can create a moving blob without corresponding to a valid compression cycle. By requiring the moving component to be inside the selected chest ROI and to follow a plausible compression rhythm, the system reduces the chance that a hand slip or unrelated background motion is counted as a deeper valid compression.

**Tier 3: synthetic fallback.** Complete detection failure is uncommon but still possible, such as when the camera is obstructed or the image becomes unusable. In that case, the system generates a sinusoidal placeholder trajectory at the region centre to preserve interface continuity. Frames handled in this way are marked invalid and excluded from all performance analyses.

**Tier switching.** To avoid rapid oscillation between detectors, switching is controlled by a delay mechanism. A new tier is accepted only when its confidence advantage persists over consecutive frames. Accepted trajectories then pass through outlier rejection, peak-preserving smoothing, and short-gap interpolation before entering the temporal buffer. These steps do not just make the signal visually smooth; they are intended to reduce false extrema that would otherwise distort the depth estimate.

### 3.3. Motion Tracking and Rate Estimation

**Signal preprocessing.** Let $y_{t}$ denote the vertical coordinate of the compression point at frame *t*. The raw trajectory is first resampled to a uniform rate by linear interpolation. It is then smoothed with a Savitzky–Golay filter whose window length adapts to the input frame rate:

(3) $$w=\min\;\left(w_{\max},\;\max\;\left(w_{\min},\;2\left\lfloor \frac{\mathrm{fps}}{2}\right\rfloor +1\right)\right).$$

This adaptation keeps the smoothing behaviour comparable across cameras with different frame rates, avoiding the need for manual tuning for each session.

**Peak–valley detection.** Compression cycles are identified under two constraints. The first imposes a minimum frame separation so that small oscillations are not misread as full compressions:

(4) $$t_{\mathrm{sep}}\geq \left\lfloor \frac{\mathrm{fps}}{f_{\min}}\right\rfloor .$$

A second condition uses a dynamic prominence threshold that adapts to the user’s recent compression depth:

(5) $$p_{\min}=\max\;\left(p_{\mathrm{base}},\;\eta \cdot \frac{\bar{d}}{k}\right),$$

where $\bar{d}$ is the rolling mean depth of recent valid cycles, *k* is the current calibration factor, and η is a fixed adaptation coefficient. A single global threshold tended to miss shallow but valid compressions and overcount noise in deeper sequences. A sustained-direction requirement further reduces double-counting near extrema, and cycles outside a physiologically plausible range are discarded.

**Compression rate.** Rate is estimated from the mean inter-valley interval over a recent window of valid cycles:

(6) $$r_{\mathrm{cpm}}=\frac{60}{\frac{1}{n-1}\sum_{i=2}^{n}\;\left(t_{v}^{\left(i\right)}-t_{v}^{\left(i-1\right)}\right)}.$$

### 3.4. Compression Depth Estimation

**Fused calibration.** Depth is estimated as

(7) $$d_{\mathrm{cm}}=\left|y_{\mathrm{valley}}-y_{\mathrm{peak}}\right|\cdot k_{\mathrm{fused}},$$

where the main difficulty lies in estimating $k_{\mathrm{fused}}$ reliably. To estimate this scale, we combine three independent scale estimates:

(8) $$k_{\mathrm{fused}}=\frac{w_{1}k_{\mathrm{def}}+w_{2}k_{\mathrm{ref}}+w_{3}k_{\mathrm{body}}}{w_{1}+w_{2}+w_{3}}.$$

Specifically, $k_{\mathrm{def}}$ is an empirical baseline calibrated from a typical webcam configuration. It is always available, but it is the least specific to the current session because it does not account for the actual camera distance or lens characteristics. To avoid participant-specific tuning, the same calibration rule was used across recordings in the reported evaluation.

$k_{\mathrm{ref}}$ comes from an object of known size placed on the manikin chest. In our study we used a standard card-sized reference. The system detects its contour, measures its span in pixels, and converts that observation into a scale estimate. The estimate is reliable only when the reference object and the compression point are at a similar distance. This assumption is reasonable when the card lies on the chest beneath the hands, but it should be kept in mind as a condition of validity for the method. If the camera or reference object is noticeably tilted, the pixel-to-centimetre factor can be biased because the reference and compression point no longer lie on the same effective image plane. For this reason, the setup interface asks the user to keep the camera fixed and to place the reference object flat on the manikin chest.

$k_{\mathrm{body}}$ uses visible shoulder landmarks. It is derived from a population-based shoulder width prior and serves as a weaker but still useful cue when the reference object is absent. Because anatomical variation is substantial, we treat this term as supportive rather than dominant in the fused estimate. In the implementation used for the study, the reference-object cue was given the largest weight when visible, the body-proportion cue was used as a secondary cue, and the default cue acted mainly as a fallback. The fusion weights were fixed before the evaluation stage and were not adjusted separately for individual participants.

When only the default cue is available, the system falls back to $k_{\mathrm{def}}$. Raw depth estimates are then filtered with a short weighted median to prevent isolated cycle-level errors from immediately propagating to the user-facing feedback.

### 3.5. CPR Metrics and Real-Time Feedback

**Setup.** Before each session, the user completes two short interactions. First, a region covering the manikin chest is selected. Second, the user marks the intended compression point at the lower sternum. This click provides the anatomical reference for all subsequent position estimates. In practice, the setup took well under a minute and was usually completed quickly enough that it did not interrupt the training flow. The camera was kept fixed throughout each recording, and participants were asked not to move the manikin after the initial calibration.

**Position error.** Given the current hand centre $\left(x_{h},y_{h}\right)$ and the sternum reference $\left(x_{c},y_{c}\right)$, we compute the position error as

(9) $$e_{\mathrm{cm}}=\sqrt{{\left(x_{h}-x_{c}\right)}^{2}+{\left(y_{h}-y_{c}\right)}^{2}}\cdot k_{\mathrm{fused}}.$$

The corresponding deviation angle

(10) $$\theta =\arctan2(y_{h}-y_{c},\;x_{h}-x_{c})$$

is quantized into directional categories for voice prompts such as “move hands up” or “shift left”.

**Feedback design.** Each metric is displayed against a colour-coded guideline band. Voice prompts are deliberately rate-limited, because continuous correction quickly becomes distracting. This decision was based on pilot observations with 12 users. When prompts fired more than once every 10 s, users became distracted and attended to the audio stream more than to the compression rhythm itself. Therefore, the system waits for a deviation to persist before issuing a cue and suppresses immediate repeats of the same message. An optional metronome channel is available for users who have difficulty maintaining rhythm. The software architecture can also accommodate haptic output, although that path was not evaluated in the present study. In the current implementation, each processing iteration includes frame acquisition, ROI cropping, landmark or motion-based tracking, cycle detection, calibration update, and feedback rendering. During pilot operation on a standard laptop CPU with a 30 fps RGB webcam, the loop remained interactive at approximately the camera frame rate, which was sufficient for real-time training feedback. The prototype was implemented with standard computer-vision libraries, including MediaPipe for hand landmarks and OpenCV-style foreground segmentation and optical flow operations. The main tier-switching and calibration rules are given above so that the processing path can be reproduced without relying on a hidden learned model.

## 4. Experiments

In this section, we will first introduce the study design including participants, sessions, and assessments. Then, we define the outcome variables explicitly. Finally, we compare our system with related work, conduct an ablation study, and present the experimental results.

### 4.1. Study Design

**Participants.** We recruited 40 adult volunteers through institutional networks and allocated them to either a System Training group or a Control group using block randomization. Experience strata were defined to distinguish participants with no prior training, lapsed certification, and current certification, and the randomization was applied within these strata to reduce baseline imbalance. Allocation was administered by an investigator who did not deliver the sessions, and informed consent was obtained before enrolment. Participants with physical conditions that prevented safe chest-compression practice were excluded.

**Sessions.** All sessions were conducted in the same laboratory with the same commercial CPR manikin, whose embedded force sensors provided the ground-truth record. The RGB camera was fixed on a tripod or stable desk position at an oblique frontal view of the manikin chest, using ordinary indoor lighting and a nominal frame rate of 30 fps. The camera position, manikin, room layout, and lighting condition were kept consistent across participants. Ground-truth compression records were synchronized with the video stream by aligning the start and end markers of each assessment recording. The synchronization was used for cycle-level agreement rather than for claiming frame-perfect correspondence between the camera stream and force-sensor trace. Participants in the System Training group first completed the VISIOCPR setup and then practised with system feedback enabled. Participants in the Control group observed an instructor demonstration and then practised with periodic verbal correction from that instructor. The same certified instructor was present in both conditions and could intervene for safety. Therefore, the difference between conditions was the mode of performance feedback rather than the physical training environment. The instructor followed the same demonstration and safety-intervention rules in both groups, but the study was not blinded because the feedback mode was visible during training.

**Assessments.** Immediately after training, all participants completed a standardized CPR assessment on the manikin while the system logged performance metrics. They also completed a questionnaire covering perceived confidence and training satisfaction. The performance assessment was repeated two weeks later so that short-term retention, and not only immediate acquisition, could be examined. Activities between the immediate assessment and follow-up were not independently monitored, so the follow-up result should be interpreted as a short-term training outcome rather than proof of durable retention.

### 4.2. Outcome Definitions

Outcome variables are defined explicitly here so that the evaluation criteria are clear and, in principle, reproducible.

**Compliance.** A compression cycle is *compliant* when rate, depth, and hand position all fall within the target range simultaneously. Rate is assessed over a local window of adjacent cycles rather than a single interval, as isolated cycle timing is too noisy to interpret on its own. Compliance is then reported as the percentage of cycles meeting this joint criterion during the assessment period. This composite endpoint is deliberately stricter than reporting the three metrics separately; it does not assume that rate, depth, and hand position are statistically independent.

**Time to Competency.** This metric denotes the elapsed training time until the participant first sustains the predefined compliance threshold. Participants who did not reach the threshold during the session were assigned the session maximum for analysis.

**Learning Efficiency.** This percentage summarizes the share of available practice opportunities in which the competency criterion was achieved by the end of training.

**Retention Score.** This is the compliance percentage observed at follow-up, using the same definition applied in the immediate post-training assessment.

### 4.3. Statistical Analysis

Continuous outcomes are reported as mean ± standard deviation. Between-group differences in continuous variables were tested with two-sided independent-samples *t* tests when distributional assumptions were acceptable; otherwise, the Mann–Whitney *U* test was used. Percentage outcomes, including Learning Efficiency, were compared using chi-square or Fisher’s exact tests as appropriate. Likert-scale confidence and satisfaction scores were analysed as ordinal outcomes using the Mann–Whitney *U* test. The significance threshold was set at p<0.05. Because several related outcomes were analysed in a modest sample, the *p* values for secondary outcomes should be interpreted as descriptive rather than as confirmatory evidence after strict multiplicity control.

### 4.4. System Comparison with Related Work

Table 1 compares VISIOCPR with representative CPR assessment systems on deployment-related attributes. A direct accuracy ranking would be misleading because the reported systems were evaluated on different manikins, under different room conditions, and with different ground-truth protocols. Therefore, the comparison focuses on sensing requirements and feedback functions rather than treating the reported accuracy values as directly interchangeable.

The comparison highlights a structural gap in the literature. Systems that report quantitative depth and provide feedback during practice usually depend on additional hardware. RGB-only systems are easier to deploy but typically stop at categorical assessment. VISIOCPR is intended to occupy that intermediate space. A stricter numerical comparison with CPR-Coach or Liu et al. is not possible from the published materials, as those datasets do not include the same type of per-cycle metric depth ground truth used here.

### 4.5. Ablation Study

To understand the contribution of each component in detail, we further conduct a module-wise ablation study. Specifically, we assessed the contribution of each component by adding it incrementally to a minimal baseline. The baseline used only pose-based keypoint detection together with the default calibration prior. Under that configuration, tracking was serviceable but not sufficiently stable for reliable coaching, and depth estimates remained too coarse for precise feedback. Adding motion-based fallback improved detection continuity. Fused calibration then reduced the scale error in depth and hand-position estimates. Finally, the feedback scheduling logic contributed the remaining gain by making the user-facing guidance more usable rather than merely more frequent.

**Figure 3:**
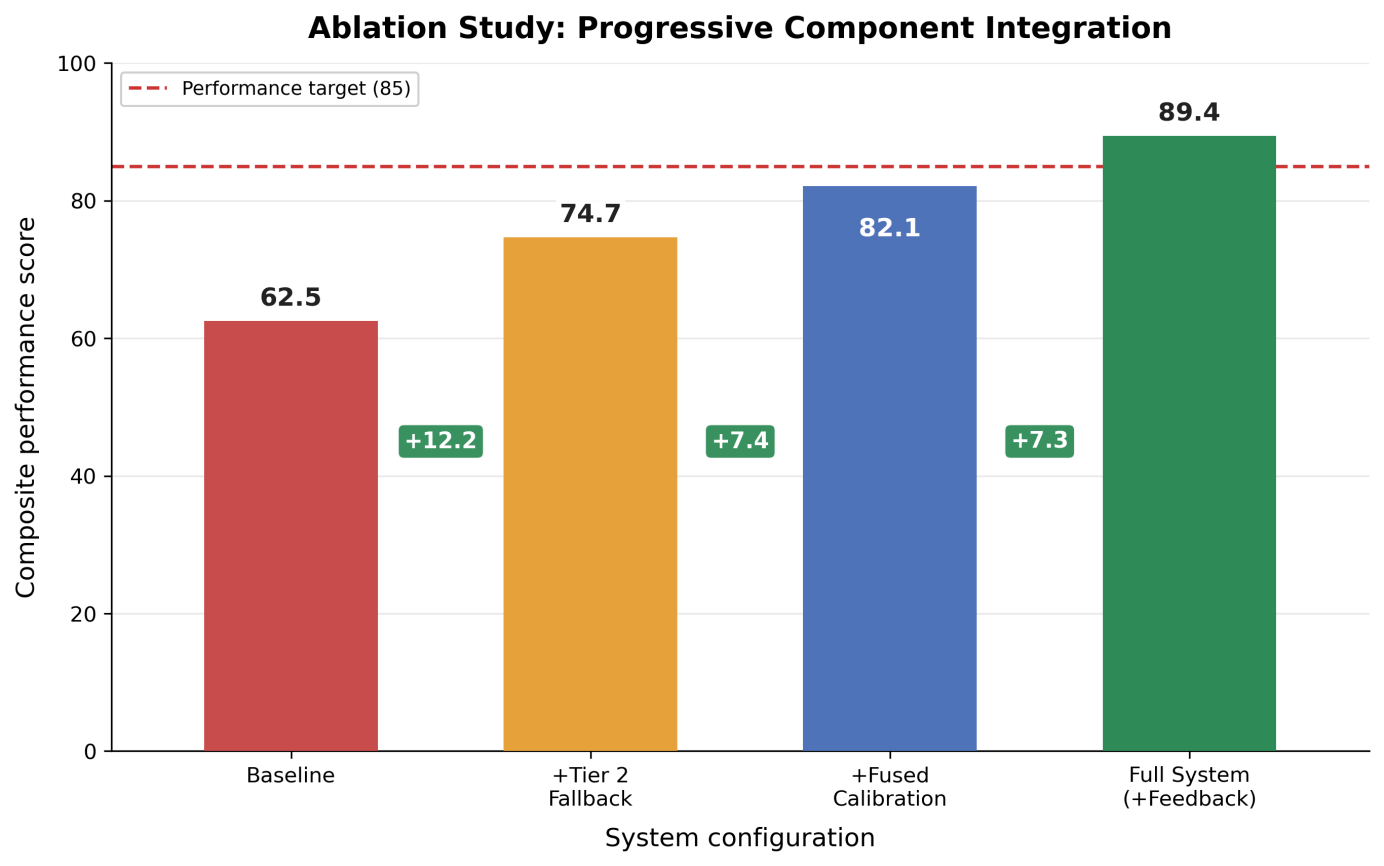
Ablation study results. Each bar shows the same composite score reported in Table 2 for one configuration, with the full system on the right. Green callouts indicate the score increase from the previous configuration after adding the corresponding component. The composite score is the unweighted average of three sub-scores: detection success rate (target 100%), depth estimation accuracy (measured as 1−normalisederror), and position error reduction relative to the no-calibration baseline. All four configurations use the same test recordings.

Table 2 and Figure 3 summarize these results with a composite score combining detection success, depth accuracy, and position error reduction. The full system outperforms the baseline in this evaluation, and the gains do not come from a single module alone. These results indicate that performance in the tested setup depends on the combination of tracking fallback, calibration fusion, and feedback scheduling. The baseline was kept runnable and used the same input recordings, but it should be understood as a minimal reference configuration rather than as a fully optimized competing system.

### 4.6. Results

**CPR performance.** Table 3 reports the immediate post-training results. Participants in the System group showed lower rate, depth, and position errors than those in the Control group. The most practically relevant outcome was joint compliance: the proportion of cycles satisfying all three criteria at once was substantially higher in the System group. We emphasize this outcome because it reflects the real training goal rather than performance on any single metric.

**Learning speed and retention.** Table 4 reports the remaining outcome measures. Participants who used VISIOCPR reached the competency threshold sooner, reported higher confidence and satisfaction, and retained a higher score at follow-up. These findings should be interpreted cautiously because the sample is limited, but they suggest that timely quantitative feedback may support faster correction during practice and better retention.

### 4.7. Discussion

The improvement in joint compliance deserves the closest attention. Viewed narrowly, the system reduced error on each individual metric; viewed more usefully, it helped participants keep rate, depth, and hand position within range at the same time. That distinction matters because CPR quality is not judged one variable at a time in actual use. A learner may maintain rhythm while drifting off the sternum, or may correct hand position while losing depth. For this reason, the joint-compliance result should be read as a strict composite training outcome, not as evidence that the three underlying errors are independent or that all learners improved uniformly on every cycle. The positional channel matters here more than it might first appear, because rate and depth feedback already exist in other forms, whereas continuous hand-position feedback is less common. This reading is consistent with prior studies showing that real-time feedback can improve CPR performance [21].

The depth results should also be interpreted relative to the target range rather than in isolation. The observed error remained smaller than the clinical tolerance band used in guideline-based training, suggesting that the estimate is useful for coaching under the tested setup. The ablation study also indicates that this accuracy does not emerge from the default prior alone, but depends on having additional calibration cues available and combining them to make the system robust to temporary cue loss.

**Limitations.** Several limitations should be kept in view. First, the sample size is adequate for detecting group differences of the size observed here, but it is not large enough for examining who benefits most under finer subgroup distinctions. Second, all recordings were collected in a controlled laboratory with a fixed camera and ordinary indoor lighting. The system has not yet been validated in homes, classrooms, outdoor spaces, or scenes with strong lighting changes and moving background objects. Third, the calibration method depends on visual assumptions that are reasonable in the present setup but are not guaranteed elsewhere, especially when the camera angle becomes steep or the reference object is not flat on the chest. Fourth, the current system monitors only a subset of CPR quality indicators. It estimates compression rate, compression depth, and hand position, but it does not directly assess chest recoil, fatigue, elbow extension, whole-body posture, breathing coordination, or interruptions between compression cycles. Fifth, the video and manikin sensor streams were aligned at the assessment-record level; residual frame-level timing error may still affect individual-cycle depth comparisons. Sixth, the training condition could not be blinded to the instructor or participants, and instructor feedback frequency was not analysed as an independent outcome. Finally, the ablation study used the same recorded test sessions for all configurations and should be interpreted as an internal component analysis rather than as an external validation of each module.

**Future directions.** Several extensions follow naturally from these limitations. One is to replace the user-placed reference object with automatic localization of stable manikin geometry. Another is to combine the present geometric method with a lightweight monocular depth network [19] to provide a fallback when the same-depth assumption weakens. A broader pose model could also extend the feedback beyond the three metrics studied here. Finally, relaxing the fixed-camera assumption would make the method easier to use in less controlled training spaces.

## 5. Conclusions

In this paper, we discuss whether a standard RGB camera could support quantitative CPR coaching without the dedicated hardware used in conventional feedback systems. The results do not suggest that a webcam is a universal substitute for instrumented manikins, but they do indicate that a useful subset of coaching functions can be recovered from monocular video if calibration, tracking, and anatomical reference selection are handled explicitly. Under the conditions tested here, the system produced depth estimates accurate enough for guideline-based feedback and was associated with better joint compliance and short-term retention than instructor-led practice alone.

The conclusion should still be read with the study boundaries in mind: the calibration procedure assumes a fairly regular viewing geometry, the sample does not support detailed subgroup claims, and the present system covers only part of what instructors watch during CPR training. Even so, the main findings suggest that low-cost RGB hardware can support a useful subset of real-time CPR coaching functions. Whether it remains reliable across broader environments and user populations is a critical issue for future research.

## Figures and Tables

**Table 1:** Attribute comparison of representative CPR assessment systems. “Quant. depth” indicates whether the system reports a continuous depth measurement in cm. “Hand pose” refers to continuous position tracking. “Real-time FB” means corrective feedback is generated during—not after—practice. Cost is the approximate additional hardware required beyond a standard PC. Boldface highlights the proposed VISIOCPR system.

System	Sensing	Quant. Depth	Hand Pose	Real-Time FB	Cost
Mini-VREM [8]	IR markers	Yes	No	Yes	∼$200+
Ruiz de Gauna et al. [5]	Accelerometer	Yes	No	Yes	∼$100
RELIVE [9]	Depth camera	Yes	No	Yes	∼$300
LISSA [13]	Depth camera	Partial	No	Yes	∼$200
CPR-Coach [10]	RGB camera	No	Yes (error class)	No	$0
Liu et al. [11]	RGB camera	No	Partial	No	$0
**VISIOCPR (ours)**	**RGB camera**	**Yes (this study)**	**Yes (continuous)**	**Yes**	**$0**

**Table 2:** Ablation results by configuration. Component increments: +12.2 (Tier 2 fallback), +7.4 (fused calibration), +7.3 (multi-modal feedback scheduling).

Configuration	Composite Score
Baseline (Tier 1 only, default calibration)	62.5
+Tier 2 motion fallback	74.7
+Fused calibration	82.1
+Multi-modal feedback scheduling (full system)	89.4

**Table 3:** CPR performance at immediate post-training assessment. Errors are mean ± SD. Compliance is the proportion of cycles meeting all three criteria simultaneously (see Section 4.2).

Metric	System	Control	*p*
Rate error (cpm)	3.2±2.1	8.7±4.3	<0.001
Depth error (cm)	0.4±0.3	1.2±0.8	<0.001
Position error (cm)	1.1±0.7	2.3±1.2	<0.01
Compliance (%)	87.5	62.3	<0.001

**Table 4:** Learning efficiency and experience outcomes. Confidence and Satisfaction are 7-point Likert scales. Retention Score is Compliance (%) at 2-week follow-up. Learning Efficiency is the proportion of participants achieving Time to Competency within 40 min.

Metric	System	Control	*p*
Time to Competency (min)	28.3±5.2	41.7±8.9	<0.001
Confidence (1–7)	6.8±0.7	5.2±1.1	<0.001
Satisfaction (1–7)	6.6±0.8	5.8±0.9	<0.01
Learning Efficiency (%)	92.5	73.8	<0.05
Retention Score	84.2±6.3	76.8±9.1	<0.01

## Data Availability

The data presented in this study are available on request from the corresponding author. Part of the data are not publicly available due to our laboratory’s confidentiality agreement and policies.
